# Dangerous liasons and hot customers for banks

**DOI:** 10.1007/s11156-022-01039-x

**Published:** 2022-02-02

**Authors:** Roy Cerqueti, Francesca Pampurini, Annagiulia Pezzola, Anna Grazia Quaranta

**Affiliations:** 1 Sapienza University of Rome, Rome, Italy; 2 London South Bank University, London, United Kingdom; 3 Università Cattolica del Sacro Cuore, Milan, Italy; 4 University of Macerata, Macerata, Italy

**Keywords:** Credit risk, Systemic risk, Financial network models, Contagion, C02, G20, G21

## Abstract

Understanding the correlation between different customers’ loss of creditworthiness is crucial to credit risk analysis. This paper describes a novel method, based on a weighted network model, in which a set of firms, customers of the same bank, represent the nodes while their links and weights derive from the total transaction amounts. We explore the contagion mechanism deriving from the transmission of the difficulties of one customer to other clients of the same bank so highlighting areas where contagion risk is higher. We use a real proprietary data set provided by a bank to illustrate the proposed approach.

## Introduction

Assessment of customer creditworthiness has always been at the heart of the banking business (Trustorff et al. 2011). It is fundamental for bank intermediaries and regulators to develop an ability to foresee bankruptcies and possess effective credit scoring systems (Shin 2010). This assures them the stability that is so important in winning the trust of the public. For any country, national economic development depends greatly on the reliability of the banking system. Most of the circumstances that produce damaging effects in banking markets derive from two factors: a) banks’ failure to observe downturns in the performance of their clients in real time, and b) how client businesses interact with each other within a given economic context.

Understanding the correlation between different clients’ loss of creditworthiness is crucial to credit risk analysis (Kahya and Panayiotis 1999; Chen and Liao 2018). Banks must include this element in their evaluation processes since it affects loan pricing, levels of capital requirements, the composition of credit portfolios and, of course, systemic risk (Heinsalu et al. 2020; Pu and Zhao 2012). Although there is no unanimous thought about how the correlation impacts credit risk, there is substantial convergence on the fact that it can be able to explain many things. Among these, for example, the level assumed, after the most recent global crisis, by non-performing loans (NPLs) within banking portfolios. Therefore, banks need to assign due weight to these correlations when they analyze the credit risk exposure of their portfolio. This in order to be able to correctly price the loan granted and, at the same time, define an appropriate diversification strategy. This is of the utmost importance for banks in general, but, in particular, for regional and interregional banks, given that their customers are characterized by the presence of even stronger interrelationships because they mainly belong to the same economic circuit and the same local community.

A criterion that takes into account the interrelationships between economic subjects also responds to the wishes of the regulatory and supervisory authorities which, as is well known, are particularly attentive to the use of models able to efficiently capture and monitor exposure to systemic risk, also for the identification of the most appropriate levels of capital requirements (Chen and Liao 2018; Eshleman and Guo 2014). Generally, the assessment of the creditworthiness of bank customers is still mainly based on approaches that assign each of them a probability of default linked to a score obtained starting from the observation and composition of various accounting and management variables^1^. Banks usually improve these models by adding hard, and soft information (Khandani et al. 2010). In a nutshell, the score is obtained by summarizing some public data, i.e. accounting data and those provided by credit bureau agencies, and of a private nature, including those relating to the bank-client commercial background. A bank that takes into account the initial analysis of a customer’s creditworthiness and in its subsequent monitoring also the dynamics of commercial interrelationships among its customers can only achieve better results in terms of forecasting any difficulties and defaults. In this respect, a bank has a huge advantage in terms of the quantity of soft information available from its customers and their mutual transactions. The analysis of the incoming and outgoing flows underlying the commercial transactions of its customers – an aspect that the standard credit default models certainly do not take into consideration – can allow a bank to better calibrate its credit policy. Indeed, through these flows it is possible to capture specific relationships and dynamics from which high-risk operations can originate as a means by which a customer’s problems are transferred to others and, consequently, to the entire local network of relationships, thus generating a chain of defaults. Here, then, is how the analysis and understanding of contagion dynamics can help a bank achieve an overall improvement in the quality of its loan portfolio and therefore also determine containment of the level of NPLs – a subject, as is well known, to which authorities are also very attentive.

This work presents a novel method related to this approach. Our analysis is based on a weighted network model (Albert and Barabási 2002; Newman 2003). We consider a set of firms that are customers of the same bank and which represent the nodes. The definition of the links and corresponding weights is based on the total transaction amounts between the firms, with a specific reference to a measure of the reaction of the customers to the losses experienced by the other ones. In particular, starting from the distress of specific customers, the work analyses the real situation (for example deriving from the existence of particular commercial agreements) that can produce negative effects only on a subset of firms connected with them; obviously, the numerousness of this subset increases when the loss entity experienced by the customers in difficulty increases. Conversely, as we will explain later, other counterparts will not suffer any contagion effect.

Then, we explore network’s resilience through the local centrality measures of the nodes, to show how firms’ relationships and cross-transactions provide insights on the presence of a contagion effect. In particular, we explore the contagion mechanism deriving from the transmission of the difficulties of one customer to other clients of the same bank, so highlighting how the difficulties of the firms of the network affect a given one.

Thus, the model presented in this work highlights the potential dangerous zones – where the risk of contagion is higher – within one bank’s customer-firms network. Model’s outcomes give an essential information, to be considered during credit risk analysis as a consequence of loan pricing. More specifically, our work aims to suggest a new methodology useful for banks to identify the customers that should be monitored more deeply and strongly. Indeed, despite their health status, some customers show a high number of reciprocal transactions with counterparties that are customers of the same bank; such interdependency can be responsible for chains of distress and shocks propagation. In detail, some highly connected customers could become extremely vulnerable because of the propagation of a spillover effect deriving from the bankruptcy of one or more of their counterparties; in the same way, some customers could become dangerous for some of their counterparties, because of their financial problems. Under the financial institutions’ perspective, both circumstances could affect (and increase) the overall level of risk of the bank’s credit portfolio.

Under the methodological perspective, we classify firms into groups on the basis of how they are dangerous and how they are vulnerable to shocks. As we will see below in detail, such a clustering procedure moves from assigning a score to the considered elements. In so doing, we are quite close to the general and broad conceptualization of the discriminant analysis (Lachenbruch and Goldstein 1979). However, the considered score comes out from the analysis of the firms as interconnected entities in a network. In doing so, we are able to fully describe the interaction of firms in situations of financial distress – which is the only mean to assess the systemic relevance of companies in terms of risk, that is exactly the target of our paper. Therefore, the complex network approach is the ground of the classification procedure; in this sense, complex networks are unavoidable in our framework.

From the analysis of the customers’ transaction dynamics, it is possible to derive the network structure that connects all these firms. At the same time, it is possible to analyse both the network’s resilience and the potential presence of critical nodes (i.e. critical firms). When needed, the bank could consider the latter information to monitor the adequacy of the pricing of the loans already granted to those customers that seemed to be extremely vulnerable and/or dangerous.

Using this knowledge, the bank can adjust its credit-granting policy over time, tightening or relaxing its criteria and rules for granting loans, thus setting appropriate loan prices, obtaining satisfactory returns and containing overall credit risk. The fact that the analysis of the network of interconnections between customers can reveal the strength and intensity of every link between them, both bilateral and multilateral, constitutes the value of the proposed approach. By means of the analysis of this network, it is in fact possible to identify customers who, potentially, can trigger a contagion effect. An important aspect that is thought to be useful to underline, at this point of the discussion, is that it is the connections between customers that determine the chain of contagion, since the latter does not depend, exclusively, on the creditworthiness of only one of them. No traditional model used for credit risk measurement purposes is able to take this specific feature into due consideration. This is why the results provided by the resilience analysis of such a network can only bring a benefit if duly integrated with the evidence deriving from the more traditionally implemented models for credit risk.

To offer a concrete example of the proposed methodology, we applied the model to a proprietary data set provided by an interregional bank in northern Italy that, referring to the EBA classification (Cernov and Urbano 2018), can be defined as a retail-oriented bank, while following the Roengpitya et al. classification (Roengpitya et al. 2014, 2017) it can be defined as a retail-funded commercial bank. Indeed, the core business of the considered bank is mainly focused on deposits collection and loans supply. The high quality of the considered empirical instance is one of the strengths of the present paper; indeed, it is rather uncommon to obtain this kind of proprietary data for scientific purposes^2^. The interregional nature of the bank is particularly useful for our purpose. Indeed, most interregional bank’s customers are headquartered in the same local reality, belong to the same community and operate within the same economic-financial environment. Consequently, the customers of a larger bank, which certainly has a more diversified loan portfolio concerning the aforementioned aspects, would have shown less intense and less strong connections than those existing between the customers of the bank we consider.

From an analysis conducted on existing literature, it does not appear that there are other studies that have already combined the results of the resilience analysis of a network with those deriving from the implementation of traditional credit risk models. It is important to point out that, the results obtained from the data provided by the bank are valid exclusively for that specific context and cannot be generalised. In any case, the analysis approach described in this work has a methodological value since it can be applied in similar situations as well as in the presence of other types of networks. In fact, this study’s results can provide an example to show how the proposed approach could be applied, how it can be useful for a bank, how network’s dynamics can be understood and carefully considered, and, therefore, which kind of strategies the bank can play out.

The remainder of the paper is organised as follows: Section 2 provides a literature review of the major studies on network analysis and its application to economic and financial topics. Sections 3 and 4 describe, respectively, the financial network model and the methodology employed. Section 5, after the description of the dataset, presents and discusses the obtained results. Section 6 concludes.

## Literature review

The risk assessment carried out by financial intermediaries and, in particular, by banks has been the object of much study over recent decades, from the perspectives of both the credit institutions themselves and a regulatory point of view. Initially, the first works dealing with the topic of credit risk were based on the famous approach proposed by Altman (Altman 1980) and then enrich it with the addition of different early warnings systems (Galindo and Tamayo 2000). These models, also known as credit scoring models, provide a number (score) representing a subject’s creditworthiness based on information regarding the subject’s characteristics and financial situation. Once the scores for all the customers to evaluate were obtained, these models ordered them according to this score, thus building a sort of ranking from the best to the worst customer. Then, each bank had the task of establishing the separation threshold (i.e. the limit score) able to divide creditworthy customers from undeserving customers in the best possible way. In this context, customers can also be classified by other (non-statistical) methods: one successful alternative approach is based on networks (West 2000). In general, the valuation models based on the Altman approach aim to estimate the default probability, or, possibly, the financial difficulty probability, of each analyzed statistical unit by combining different information of a mainly financial nature measured via appropriate proxies. Naturally, models of this type can offer a statistical unit’s default probability estimation, but they are not able to identify the actual situations that cause the customer’s financial distress (Chen and Huang 2003).

For this reason, to overcome this limit, credit risk assessment models were subsequently introduced, able of providing information not only on the manifestation of the default event, but also on the causes that can generate it, drawing inspiration from the well-known theory of Capital Asset Pricing Model (CAPM). The novelty introduced with these models is the ability to measure – as well as to take into account – any correlations existing between different statistical units, especially when a default occurs; in this way, it becomes possible to study the correlation between the events that characterize the credit risk. The first of these models was proposed by Merton (1974): in this model, the problem of credit risk correlation was tackled by assuming that stochastic processes observable for the assets of two companies are correlated. Other models within this strand of literature have focused particularly on the strong contagion effects between bankrupt firms (Jorion and Zhang 2007; Horst 2007; Hatchett and Kuehn 2009) and have tried to incorporate new types of risk factors (Goldstein et al. 2002; Duffie et al. 2009; Giesecke 2004; Giesecke and Weber 2004, 2006; Jarrow and Yu 2001; Schönbucher and Schubert 2001). Although this new class of models for the study of credit risk has immediately shown itself to have a good predictive capacity, their use has been rather limited. This is due to the circumstance that, to implement such models, it is necessary to have information on a wide range of easily available market variables for all the firms listed on regulated markets. Of course, the same information is completely non-existent in the case of unlisted companies, while, as it is well known, most of the commercial banks’ credit portfolio is made up of precisely this type of company and therefore market models cannot be used.

A further aspect that, over time, has contributed to renewing and improving models for evaluating credit risk is the possibility of including in the evaluation items that the model can manage, also the so-called soft information. The bank-client relationship is a typical long-term relationship based on fiduciary dynamics and repeated transactions; valuation models capable of incorporating, in addition to the normal financial variables, additional proxies representing the behavior of customers and the type of relationship with their bank have proven to be better than previous ones^3^. Actually, this information is used by banks for a variety of purposes, not only to assess creditworthiness, but also to implement cross-selling activities and to carry out customer satisfaction evaluations (O’Brien et al. 2002). The diffusion of techniques and models capable of dealing with this new class of variables has stimulated the birth of new research lines (Setiono et al. 1998) focused on behavioral scoring models, statistical classification techniques, neural networks and data mining (Hand 1981; Johnson and Wichern 1998; Lacher et al. 1995). All these techniques are generally related to machine learning, i.e. those methodologies used for the analysis and solution of problems characterized by the presence of a considerable-sized data set that, to be properly studied, requires specific algorithms (Khandani et al. 2010). The new frontier of credit risk research is precisely based on the application of innovative machine learning techniques to models for the assessment of creditworthiness since through these techniques it is possible to use a large number of input variables representative of both qualitative and quantitative aspects (Petropoulos et al. 2019). Numerous studies have recently demonstrated the predictive superiority of machine learning techniques^4^ over more traditional models for evaluating defaults Addo et al. (2018).

The risk analysis and measurement techniques evolution has stimulated the diffusion of new models aimed not only at studying credit risk, but also systemic risk; indeed, the two aspects have often been jointly analysed precisely because of their strong interconnection and interdependence (Martinez-Jaramillo and Battiston 2020). Analyses of this type presuppose the need to analyze not only a very large number of qualitative and quantitative variables, but also the different dynamics with which they interact, triggering different effects from time to time. This is possible only thanks to the use of advanced machine learning techniques such as those implemented by Petrone and Latora (2018) and Giudici et al. (2019). The first two authors focus on the measurement of the systemic risk that arises from the reciprocal relationships between financial intermediaries. The traditional credit risk models are combined with new techniques capable of adequately representing the relational dynamics between the banks that are in the market, in particular the European Global Systemically Important Banks. The second paper proposes the combined use of traditional modeling and similarity networks to improve the accuracy of credit risk estimation. Specifically, the authors use network analysis to extract new explanatory variables that can be used to enrich the credit risk estimation model. The last work is an interesting review of the most recent scientific works that deal with the use of network models in financial applications.

Most of the works that deal with systemic risk study through network analysis are focused on the interbank market. After the pioneering work of Allen and Gale (2000) focused on the mechanism of financial contagion, subsequent research tried to offer a deeper analysis of the structure of interbank market network highlighting many factors that could contribute to switching on a spillover effect such as liquidity crises, incomplete contracting, unsecured claims and repo activity, similarities in investment strategies (Castiglionesi 2007; Gai et al. 2011; Allen and Carletti 2013; Chinazzi and Fagiolo 2015; Aymanns and Georg 2015; Pino and Sharma 2019). Another interesting and recent work by Biswas and Gómez (2018) propose a model in which banks are exposed to the risk of contagion through their portfolio of loans, showing how a solvency problem in one bank can be transmitted to another if they lend to the same borrower.

Really, the possibility of having large amounts of information and the ability to process these data effectively and efficiently is undoubtedly the result of the technological evolution that has characterized the last decades and that has allowed machine learning techniques to be used in all scientific disciplines. Furthermore, since the global crisis, central banks have undertaken many data-based/statistical analyses aimed at supporting and furthering their supervisory and monetary policy functions.^5^ Of course, the availability of an extraordinarily large set of information is not in itself a sufficient condition to improve the Central Banks’ supervision work; indeed, it is also essential to implement robust data mining processes and advanced analytical techniques in order to exploit all the information power of the data set available. Conventional statistical and econometric methods cannot capture these datasets’ multidimensional aspects, hence leading to a preference for a framework based on advanced machine learning techniques and complex networks.

The issue of data is considered the main problem in the field of network-based analysis on systemic risk. It is not easy to collect all the information needed to describe the structure of the network that characterizes a particular environment, such as, for example, the interbank market. Otherwise, this information is essential to understand the dynamics of the reciprocal relationships between nodes and, consequently, the dynamics of potential spillover mechanisms. Some authors tried to overcome this problem by employing only publicly available information about the characteristics of every single subject (node), such as information coming from financial statements; based on these data, they tried to infer the structure of the network (Glasserman and Young 2015) and the reaction dynamics after different kinds of shocks, hence highlighting the progression of the contagion (Gençcay et al. 2020). In this work, we employ a similar approach based on the information about every node to study the dynamics of systemic risk and contagion in a network represented by the reciprocal relationships between a single bank’s customers. To the best of our knowledge, this is the first work that offers this particular approach to combine the analysis of credit risk and systemic risk in a network based on some reciprocal financial relationships between nodes.

## The financial network model

We consider the customers of a bank as the nodes of a directed network. We collect them in the set of the nodes $V=\{1, \dots , n\}$.

Given $i, j \in V$, we consider the yearly financial flow from a customer *i* to a customer *j*. Such a flow is an in-flow of *j* and an out-flow of *i*. If such a flow is positive, then we have a directed arc from *i* to *j*. The weight of this arc is $w_{ij}$, and it is measured through the entity of the financial flow from *i* to *j*. When the financial flow is null, then there is not a directed arc from *i* to *j*. Generally, $w_{ij} \ne w_{ji}$. Moreover, we do not consider loops, so that we impose $w_{ii}=0$, for each $i \in V$.

The terms *w*’s are collected in the weighted adjacency matrix of the network, namely *W*. Such a matrix describes the inter-flows among the customers of the bank. By definition, the reading of *W* is then enough to state the existence of an arc from a node to another one. The directed network of customers and financial inter-flows among them is then ${\mathcal {N}}=(V,W)$.

Network ${\mathcal {N}}$ is the starting point of our analysis, but it is not the core of our interest. Indeed, ${\mathcal {N}}$ induces a new directed network ${\mathcal {N}}_R$ sharing the same nodes of ${\mathcal {N}}$ but with different weighted connections. The meaning of the subscript *R* will be clear soon. Starting from the distress of a specific customer *i*, it is possible to analyze the recurring situation in which the aforementioned distress can produce negative effects only on a subset of firms connected with it; obviously, the numerousness of this subset increases when the loss entity experienced by the customer in difficulty increases. Conversely, other counterparts will not suffer any contagion effect. So, we fix a node $i \in V$ and assume that a percentage $\alpha \in (0,1)$ of the all out-flows of *i* is removed. Such a removal is distributed to a particular subset of the nodes receiving a financial flow from *i*.

The set ${\mathcal {I}}_i(\alpha )$ collects the customers – connected with *i* through an in-flow – that are vulnerable to a financial distress of *i*.

Clearly, the effect of this removal on the total amount of the in-flows of *j* depends on the selected node *j*. We enter the details.

We define the total in-flow of a node *j* as

1 $$\begin{aligned} \phi (j)=\sum _{k \ne j} w_{kj}. \end{aligned}$$

In the same way, we can define the total out-flow of a node *i*.

When we remove a percentage $\alpha \in (0,1)$ of the all the out-flows of node *i*, then the total in-flow of *j* in (1) becomes

2 $$\begin{aligned} \phi ^{(i; \alpha )}(j)= \left\{ \begin{array}{ll} \sum _{k \ne i} w_{kj}+w_{ij}(1-\alpha ) &{} \hbox {if }j \in {\mathcal {I}}_i(\alpha ) \\ \sum _{k \in V} w_{kj}, &{} \hbox {otherwise} \\ \end{array} \right. \end{aligned}$$

Of course, $0 \le \phi ^{(i; \alpha )}(j) \le \phi (j)$, for each $i,j \in V$ and $\alpha \in (0,1)$, and $j \notin {\mathcal {I}}_i(\alpha )$ implies that $\phi ^{(i; \alpha )}(j)= \phi (j)$. Moreover, fixed $\alpha \in (0,1)$, the deviation between $\phi ^{(i; \alpha )}(j)$ and $\phi (j)$ depends on *i* and *j*; such a deviation explains the relevance of the loss of a percentage $\alpha$ of the financial out-flows of *i* for the overall financial in-flows of *j*. If such a deviation is of large size, then $j \in {\mathcal {I}}_i(\alpha )$ and a large part of the in-flows of *j* are due to customer *i*; differently, a small deviation means that *j* is not remarkably affected from a shrink of the total out-flows of *i*, even if $j \in {\mathcal {I}}_i(\alpha )$. We formalize this remark.

Given $\alpha \in (0,1)$ and $i, j \in V$ with $i \ne j$, we define the indicator

3 $$\begin{aligned} \lambda _{ij}(\alpha )=1-\frac{\phi ^{(i; \alpha )}(j)}{\phi (j)}. \end{aligned}$$

By definition, $\lambda _{ij}(\alpha ) \in [0,1]$. Trivially, $\lambda _{ij}(\alpha )=0$ when $j \notin {\mathcal {I}}_i(\alpha )$. Otherwise, as the value of $\lambda _{ij}(\alpha )$ approaches one (zero), then a removal of a percentage $\alpha$ of the total out-flow of *i* has a severe (weak) impact on the total in-flow of *j*. Moreover, since $w_{ii}=0$, then (1), (2) and (3) gives that $\lambda _{ii}(\alpha )=0$, for each $i \in V$ and $\alpha \in (0,1)$.

We collect all the $\lambda (\alpha )$’s in a squared matrix of order *n*, namely $\Lambda (\alpha )$. Evidently, also matrix $\Lambda (\alpha )$ is generally not symmetric. Such a matrix represents the weighted adjacency matrix of the above-mentioned directed network ${\mathcal {N}}_R(\alpha )=(V, \Lambda (\alpha ))$.

By construction, network ${\mathcal {N}}_R(\alpha )$ gives a clear vision of the way in which a loss of the flows coming out from a node affects the entire network, with reference also to the set ${\mathcal {I}}_i(\alpha )$. Moreover, the detailed analysis of the individual elements or of the rows and the columns of the matrix $\Lambda (\alpha )$ allows to provide a specific information on the financial inter-flows among the single customers of the bank. Therefore, ${\mathcal {N}}_R(\alpha )$ gives relevant insights on the financial vulnerability of the system generated by the customers of the bank, when the connections through their inter-flows are considered. The subscript *R* stand here for *Risk*. The loss of a percentage $\alpha$ of the total out-flow of a node is assumed to play the role of an exogenous shock; its propagation over the nodes is captured by the related terms in matrix $\Lambda (\alpha )$.

## Methodology

As stated before, the usefulness of the proposed approach is to explain what happens between the bank’s customer firms in terms of cash flows exchanged because of their commercial relationships and, therefore, how the bank can also use this knowledge to manage the risk of contagion deriving from mutual connections between customers. In this way, the bank can also consider this information when monitoring the credit granted to each customer as well as when designing its strategies aimed at managing credit risk.

With more details, the dynamics of mutual transactions between its customers can help a bank to bring out the structure of the network that connects them, to evaluate the overall resilience of this network as well as the presence of particularly critical nodes that need to be monitored more thoroughly. This last information will be useful to the bank both for reshaping, if necessary, the conditions underlying the credit already granted to those customers who seem vulnerable (because of the propagation of a spillover effect deriving from the bankruptcy of one or more of their counterparties) and/or dangerous (for some of their counterparties, because of their own financial problems), and, at the same time, for assessing the adequacy of the provisions made to cover the risk of the loan portfolio and, ultimately, for adjusting the capital levels required by the supervisory authorities.

The analysis of the network ${\mathcal {N}}_R(\alpha )$ will be carried out under different perspectives, by starting from an empirical instance of high-quality real data. Two different approaches are adopted for the analysis: first, the study of the main descriptive statistics of the elements of $\Lambda (\alpha )$, second, the exploration of some relevant measures of the network ${\mathcal {N}}_R(\alpha )$, to be considered either at the individual nodes level as well as at the overall system level. The former approach allows to understand in depth the reaction of the customers of the bank to the loss of the in-flow from another customer under a purely data science point of view, without taking into consideration the interconnections among the involved elements. The latter one serves for having a clear idea of the contagion, by including also the topological structure of the network and the strength of the interconnections.

A large number of scenarios are considered, by taking into account several values of the parameter $\alpha$ that varies between 0.1 and 0.9 with a step equal to 0.1. In doing so, we are able to discuss the obtained findings in the light of the entity of the occurred exogeneous shock.

For the network measures, we consider the following quantities:

- in-degree and out-degree of a node *i*, which are defined, respectively, by
  4 $$\begin{aligned} d^{in}_i(\alpha )= \sum _{j \in V} \lambda _{ji}(\alpha ) \qquad d^{out}_i(\alpha )= \sum _{j \in V} \lambda _{ij}(\alpha ). \end{aligned}$$
  These measures provide a quick view of the relevance of the individual customers of the bank in terms of their vulnerability against a loss of percentage $\alpha$ of the nodes in-connected to it (case of the in-degree) and in terms of the (dangerous) impact of their own losses on the other customers of the network (case of the out-degree);
- in- and out-clustering coefficient of a node *i*, defined, respectively, by
  5 $$\begin{aligned}&C^{in}_{i}(\alpha )= \frac{\sum _{j,k \in V\setminus \{i\}} [\lambda _{ij}(\alpha )\lambda _{jk}(\alpha )\lambda _{ik}(\alpha )]^{1/3}}{(n-1)(n-2)} \nonumber \\&C^{out}_{i}(\alpha )= \frac{\sum _{j,k \in V\setminus \{i\}} [\lambda _{ji}(\alpha )\lambda _{jk}(\alpha )\lambda _{ki}(\alpha )]^{1/3}}{(n-1)(n-2)}. \end{aligned}$$
  The definition of the clustering coefficients in (5) is an adaptation of the one of Onnela (Onnela et al. 2005; Saramäki et al. 2007) in the light of the version of such a concept for directed networks proposed and explored in Clemente and Grassi (2018), Cerqueti et al. (2021). The clustering coefficient synthesizes the community structure around the nodes of the network ${\mathcal {N}}_R$ when one considers the effect on the nodes of the loss of one node (out-clustering coefficient) or the effect on one node of the losses of the other nodes (in-clustering coefficient). By definition, $C^{in}_{i}(\alpha ), C^{out}_{i}(\alpha ) \in [0,1]$. As the value of the clustering coefficient approaches one (zero), then the community structure around the considered node becomes stronger (weaker). Here, a strong community structure for the in-clustering coefficient $C^{in}_{i}(\alpha )$ means a highly vulnerable node *i* in presence of the losses of a percentage $\alpha$ of the in-flows from the other nodes; differently, a strong community structure for the out-clustering coefficient $C^{out}_{i}(\alpha )$ means that the overall system of the customers of the bank is highly vulnerable to the loss of a percentage $\alpha$ of the in-flows from *i*.

Starting from the arguments above, it is clear the connection between the clustering coefficients and the resilience of the bank’s customers network.^6^ In our specific context, the bank’s customer firms network is highly resilient when the difficulties of a specific customer will not be such as to compromise the balance of the entire network, since the contagion effect towards the other elements of the latter, is very limited thanks to the weak connection between the different nodes (Glasserman and Young 2015; Edirisinghe et al. 2015). Therefore, in line with the definition of the clustering coefficients in (5), low values of the $C^{in}$’s and $C^{out}$’s are associated to a highly resilient network. Conversely, high values of the clustering coefficients suggest a scarcely resilient network.

It is crucial to point out that the clustering coefficients are not enough to fully describe the vulnerability of the network’s nodes. Indeed, there is not a straightforward relationship between the value of the clustering coefficient and the degree of the related nodes. As an example, a given node *i* can have a high value of $C^{in}_{i}(\alpha )$ but a low in-degree $d^{in}_{i}(\alpha )$. In this circumstance, even if the node *i* is highly vulnerable when its adjacents experience a loss $\alpha$, the number of the adjacents is so low that its impact on the entire network’s resilience is negligible. The consequence of this argument is that clustering coefficients and degrees have to be jointly evaluated, for having a complete view of the status of the considered network. We consider that a high value of the degrees are associated to potential properties of the nodes; such properties become real only if also the clustering coefficients have a high value. More specifically, we cluster the bank’s customers into four groups, as follows:

- *Group 1:* customers that are effectively vulnerable and really dangerous. A node *i* belongs to this group when it has a high value of $C^{in}_{i}(\alpha )$ and $d^{in}_{i}(\alpha )$ – hence, *i* is effectively vulnerable to the losses of a percentage $\alpha$ of the other nodes of the network – and, at the same time, a high value of $C^{out}_{i}(\alpha )$ and $d^{out}_{i}(\alpha )$ – hence, *i* is really dangerous for the others when it experiences a loss of percentage $\alpha$.
- *Group 2:* customers that are only effectively vulnerable. A node *i* belongs to this group when it is not in Group 1, but it has a high value of $C^{in}_{i}(\alpha )$ and $d^{in}_{i}(\alpha )$.
- *Group 3:* customers that are only really dangerous. In this case, *i* does not belong to Group 1, but $C^{out}_{i}(\alpha )$ and $d^{out}_{i}(\alpha )$ have high values.
- *Group 4:* customers that are not really dangerous nor effectively vulnerable. This group collects all the nodes which do not belong to the other groups.

Since the clustering coefficients and degrees distributions are skewed^7^, in the empirical experiments we will say that the values of these variables are “high” when they are higher than the median one.

For each of these groups, based on the information made available by the bank, further analyses will be carried out trying to understand if it is possible to relate the belonging to a specific group with other qualitative and quantitative characteristics captured by the data coming from the balance sheets and from the bank’s private information. This is to verify the possible existence of recurrences and regularities in each of the targeted groups and therefore to bring out specific aspects that could reasonably be subject to further monitoring by the bank.

## Empirical results

The dataset used to illustrate the proposed approach refers to the year 2020 and to the group of customer firms of an Italian interregional commercial bank operating in northern Italy. They are all small-medium enterprises (SMEs) characterized by the highest number of reciprocal transactions (i.e. the incoming and outgoing monetary flows).

This specific group does not include all the larger firms that are customers of the bank since most of them do not have particularly intense relationships with the others. In line with the theoretical model, we build the network ${\mathcal {N}}$ by considering the entity of the reciprocal transactions. So, network ${\mathcal {N}}$ consists of $n = 250$ nodes, connected to each other through $L = 11.354$ directed links.

Moreover, we provide information on the economic sector, legal form and turnover and on some of the most critical balance sheet indicators (chosen by the bank itself) used during the creditworthiness investigation for each customer.

**Fig. 1:**
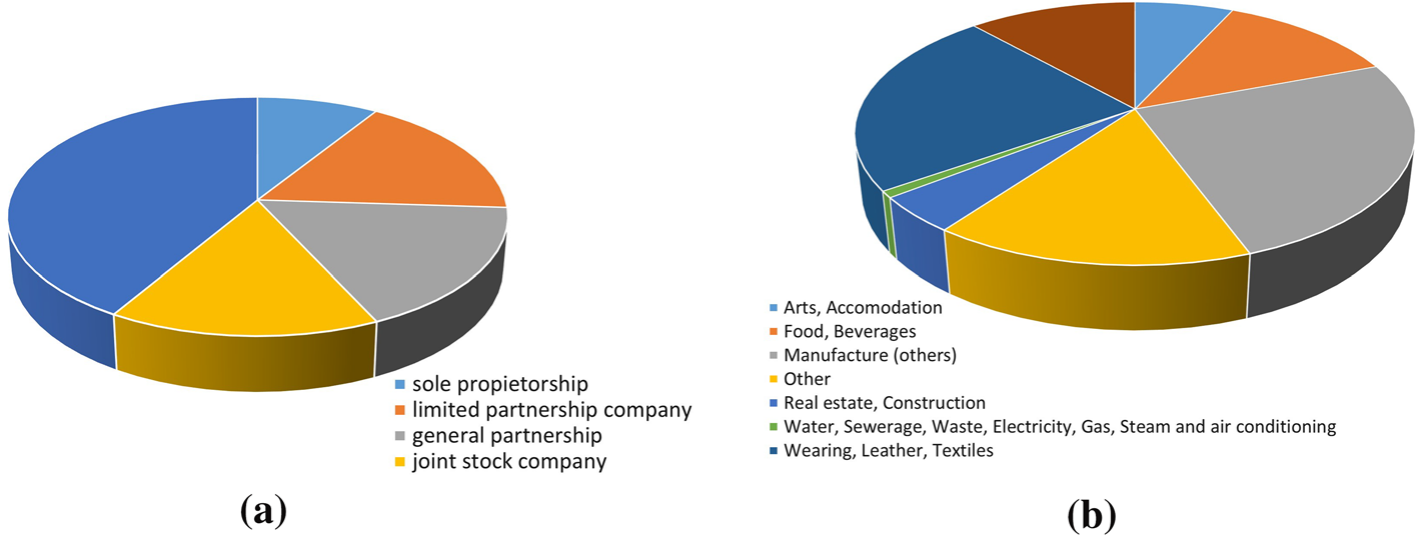
Frequency distributions of legal form (**a**) and economic sector (**b**) of the customer firms included in the dataset

Table 1 shows the main descriptive statistics of the considered balance sheet variables, while Figure 1 illustrates the frequency distributions of the bank’s customers legal form and economic sector.

**Table 1:** Descriptive statistics of the balance sheet variables considered in the analysis$^*$

	Mean	Median	St.Dev.	cv	Min	Max	Skewness	Kurtosis	Shannon Entropy
Flows (€)	8.360	4.830	10.359	1,24	20	57.483	2,47	6,21	0,987
Sales (€)	20.507.710	14.265.759	18.976.335	0,93	61.462	66.954.887	0,6	−1,04	1
Leverage ratio$$^{**}$$	1,28	1,01	0,91	0,72	0,022	3,19	0,58	−0,92	0,995
EBITDA/Sales	0,10	0,094	0,058	0,58	0,0005	0,222	0,21	−0,92	0,988
ROE	4,71	2,99	4,37	0,93	−2,16	13,8	0,57	−1,01	0,96
ROA	2,62	1,15	3,32	1,27	−1,16	14,36	1,46	1,37	1
ROI	3,64	2,32	3,58	0,98	−1,54	14,97	0,81	−0,30	1
Credit drawn/Credit granted	56	53	19	0,34	30	115	1,05	0,76	0,95
Liquidity ratio$$^{***}$$	0,74	0,76	0,28	0,38	0,1	1,2	0,38	−0,33	0,96
Default probability	0,039	0,03	0,03	0,78	0,01	0,15	1,93	3,6	0,78

$^*$ Flows and Sales are measured by Euros, while the other variables are percentage.

$$^{**}$$ The Leverage Ratio is obtained as Debt to Total Assets.

$$^{***}$$ The Liquidity Ratio is obtained as Liquid Assets to Total Assets.

**Fig. 2:**
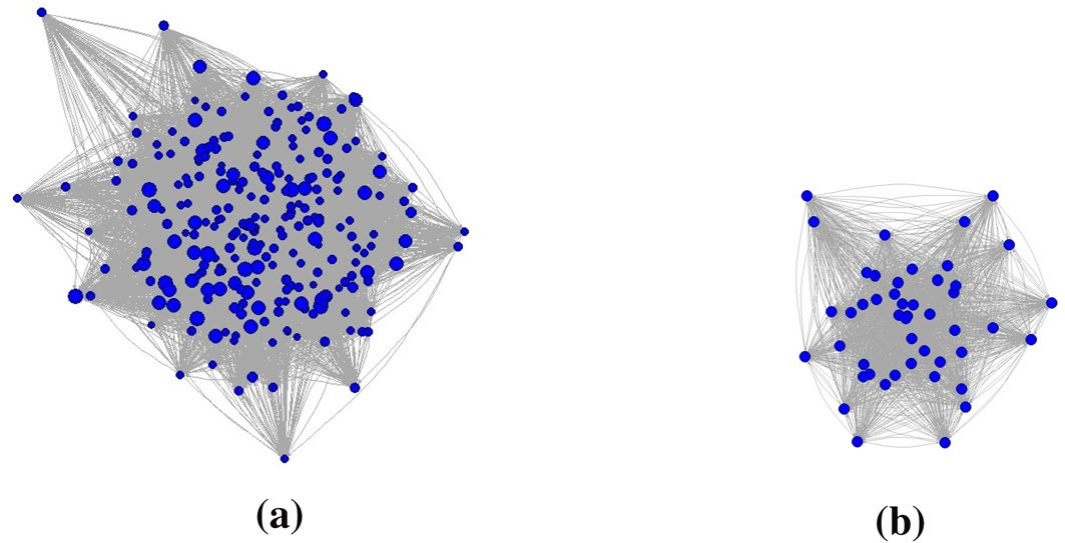
Representation of network ${\mathcal {N}}$. Panel (**a**) contains the whole network, while panel (**b**) describes the subnetwork with the nodes having the highest number of connections

Figure 2 represents the network ${\mathcal {N}}$ (i.e., as mentioned before, the incoming and outgoing monetary flows, and, therefore, the monetary values of the commercial relationships in place) between bank’s customers (to make the graph more visible, the arrows were not weighted with respect to the amount of flow exchanged). In particular, Figure 2a represents the whole network, while Figure 2b offers a zoom of the nodes with the highest connections number.

The size of the nodes has been calibrated with respect to the total amount of flows (indeed, it is the algebraic sum of incoming flows and outgoing flows). This information is already very important by itself: the larger the node’s size, the more monitoring is needed. This because the size immediately gives an idea of the strength (and therefore of the importance) of each node with respect to the others as the size is able to summarise the total number - and therefore the total amount - of transactions in which every customer is involved. The graph layout used is the Kamada-Kawai layout algorithm. It places the vertices on the plane, or in the 3d space, based on a physical model of springs. The largest nodes are placed in the centre of the network.

With reference to the construction of matrices $\lambda _{ij}(\alpha )$, we introduce a squared matrix of order *n* of realizations of a dummy variable; in particular, in this matrix, a value equal to 1 indicates that the corresponding node *j* belongs to the subset ${\mathcal {I}}_i(\alpha )$ – and so it is affected by the shock on the flow $w_{ij}$ coming from node *i* – while such a value is 0 otherwise. In so doing, we reproduce the real dynamics of the commercial transactions between firms – that are influenced by the different agreements about the timing for the completion of each trade’s payment – by specifically respecting the hypothesis that only for a subset ${\mathcal {I}}_i(\alpha ) \subseteq V$ of receiving nodes the flow becomes $w_{ij}(1-\alpha )$, while, for the remaining nodes the flows are unchanged.

We proceed by simulation for building such a matrix. Indeed, we do not have specific information from the bank about the commercial agreements between the customers – i.e., we are not in the position of empirically selecting in our case the firms that are influenced by the shocks of a given firm. This information is strictly confidential, but it is needless to say that banks usually have such relevant information to use our methodological framework by employing high-quality empirical data. For this lack of information, we implement a scenario analysis based on the variation of the percentage $\alpha \in (0, 1)$ of the not influenced nodes. Specifically, given firm *i*, we randomly select a percentage $\alpha$ of the firms of the sample affected by the shock of *i*. We start with the case $\alpha =0.1$ – that is the less worrying scenario since it is associated with the lighter shock – hence building a matrix with 10% of ones and the remaining 90% of elements as zeros. The selection of the ones and zeros follows a purely random process. After implementing the analysis in this first case, we increase the percentage to $\alpha =0.2$ and redo the calculation. In increasing $\alpha$, we start from the matrix built in the case $\alpha =0.1$ and replace some zeros randomly with ones so that the final matrix has 20% ones and 80% zeros. Then, we proceed iteratively by increasing $\alpha$ by 0.1 at each iteration, till $\alpha =0.9$. In increasing $\alpha$, we simulate the fact that when the shock increases each firm *i* meets much more difficulties to fulfill its commitments; thus, an increasing number of counterparts joins the group ${\mathcal {I}}_i(\alpha ) \subseteq V$.

In Table 2, we show the main descriptive statistics of matrices $\Lambda _{in}(\alpha )$ and $\Lambda _{out}(\alpha )$ obtained from the formulas (1), (2) and (3).

**Table 2:** Descriptive statistics of all the values contained in matrices $\Lambda _{in}(\alpha )$ and $\Lambda _{out}(\alpha )$. On the columns, we have the different values of the $\alpha$’s – the considered values are reported in bold in the first row

$\Lambda _{in}$	0,1	0,2	0,3	0,4	0,5	0,6	0,7	0,8	0,9
Mean	0,0153	0,0154	0,0158	0,0163	0,0172	0,0181	0,0192	0,0202	0,0214
St.dev	0,0700	0,0705	0,0709	0,0730	0,0760	0,0795	0,0822	0,0844	0,0879
Max	1,1684	1,1889	1,2094	1,1723	1,5679	1,8352	1,8648	1,8944	1,9240
Min	1,74E-04	1,74E-04	1,75E-04	1,75E-04	1,75E-04	1,75E-04	1,75E-04	2,10E-04	2,13E-04
Skewness	4,3788	4,4175	4,0832	4,1367	4,5121	4,8882	4,6672	4,4861	4,4028
Kurtosis	36,4624	37,1143	28,7188	30,1681	44,5999	60,3760	55,2578	51,4912	48,7824

$\Lambda _{out}$	0,1	0,2	0,3	0,4	0,5	0,6	0,7	0,8	0,9
Mean	0,0155	0,0155	0,0159	0,0165	0,0173	0,0183	0,0191	0,0203	0,0215
St.dev	0,0695	0,0699	0,0707	0,0732	0,0766	0,0798	0,0819	0,0849	0,0891
Max	2,1084	2,1454	2,1824	2,2194	2,2564	2,2933	2,3303	2,3608	2,4043
Min	3,89E+09	3,89E+09	2,86E+09	3,89E+09	3,89E+09	3,89E+09	5,90E+09	5,90E+09	6,90E+09
Skewness	9,6692	9,6274	9,5875	9,3616	9,4675	9,2430	9,0641	8,5940	8,5133
Kurtosis	7,6919	7,6007	7,8453	8,1035	8,0354	7,5954	7,9256	7,7954	7,8628

**Fig. 3:**
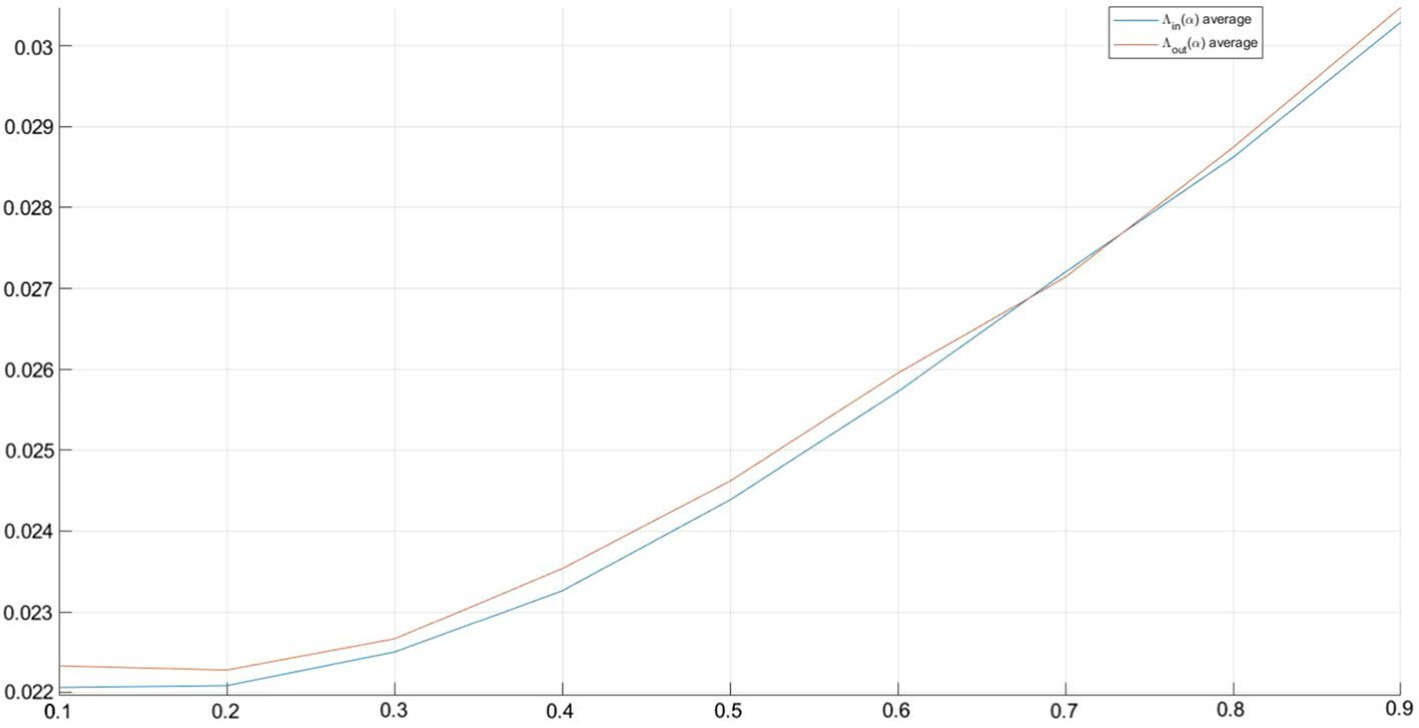
$$\Lambda ^{in}(\alpha )$$ and $$\Lambda ^{out}(\alpha )$$ means

Summing up, it is possible to note that^8^:

- both in $\Lambda _{in}(\alpha )$ and $\Lambda _{out}(\alpha )$ the mean increases (see Fig. 3), slowly, when $\alpha$ increases. Similarly, in both cases, the variability increases, slowly, as $\alpha$ increases, and it is always consistently high;
- maximum values in $\Lambda _{out}(\alpha )$ are greater than those in $\Lambda _{in}(\alpha )$, while the opposite result emerges for minimum values in $\Lambda _{in}(\alpha )$ and $\Lambda _{out}(\alpha )$;
- the matrixes $\Lambda _{in}(\alpha )$ show a lower skewness and a higher kurtosis with respect to the matrixes $\Lambda _{out}(\alpha )$. Moreover, skewness and kurtosis values remain stable when $\alpha$ changes.

Although it is unlikely that this circumstance will occur, for completeness the borderline case in which all nodes *j* belong to subset ${\mathcal {I}}$ was also analyzed.

Summing up, it was possible to observe (data available upon request) that (i) the mean, median, standard deviation, maximum and minimum values change for a constant amount when $\alpha$ changes; (ii) the mean values are equal for all matrices $\Lambda _{in}(\alpha )$ and $\Lambda _{out}(\alpha )$; (iii) maximum values in $\Lambda _{out}(\alpha )$ are greater than those in $\Lambda _{in}(\alpha )$, while the opposite result emerges for minimum values in $\Lambda _{in}(\alpha )$ and $\Lambda _{out}(\alpha )$; (iv) skewness and kurtosis are both greater than zero in relation to $\Lambda _{in}(\alpha )$ and $\Lambda _{out}(\alpha )$ matrices and, obviously, their values are constant for every shock size.

With reference to the calculation of $d^{in} (\alpha )$ and $d^{out} (\alpha )$, we observe that a customer that is potentially more vulnerable (high $d^{in}$ value) and/or more dangerous for the others (high $d^{out}$ value) maintains this characteristic for each value of $\alpha$. The same occurs in the borderline case analyzed.

**Fig. 4:**
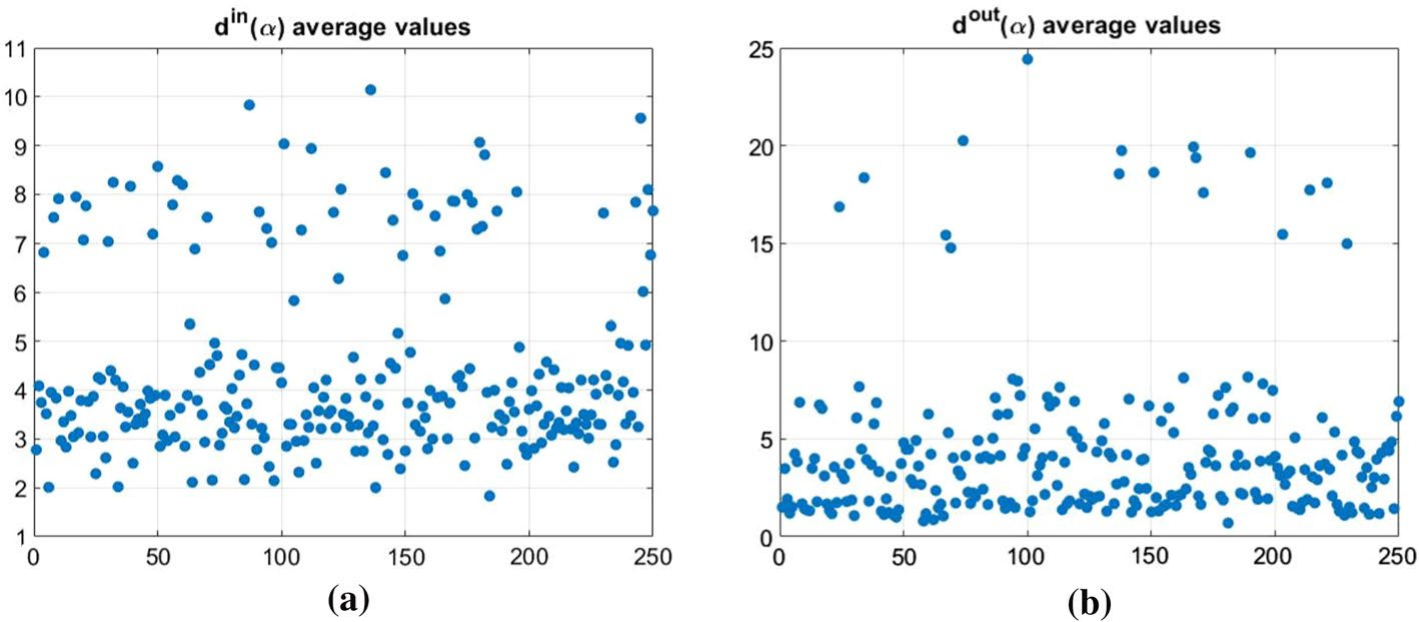
$\mathbf{d }^{in}(\alpha )$ and $\mathbf{d }^{out}(\alpha )$ mean values related to each customer firm

Stating the above, we synthesize the different in-degrees and out-degrees through the means of $d^{in}(\alpha )$ and $d^{out}(\alpha )$ over $\alpha =0.1, 0.2, \cdots , 0.9$, for each customer (see Fig. 4).

**Fig. 5:**
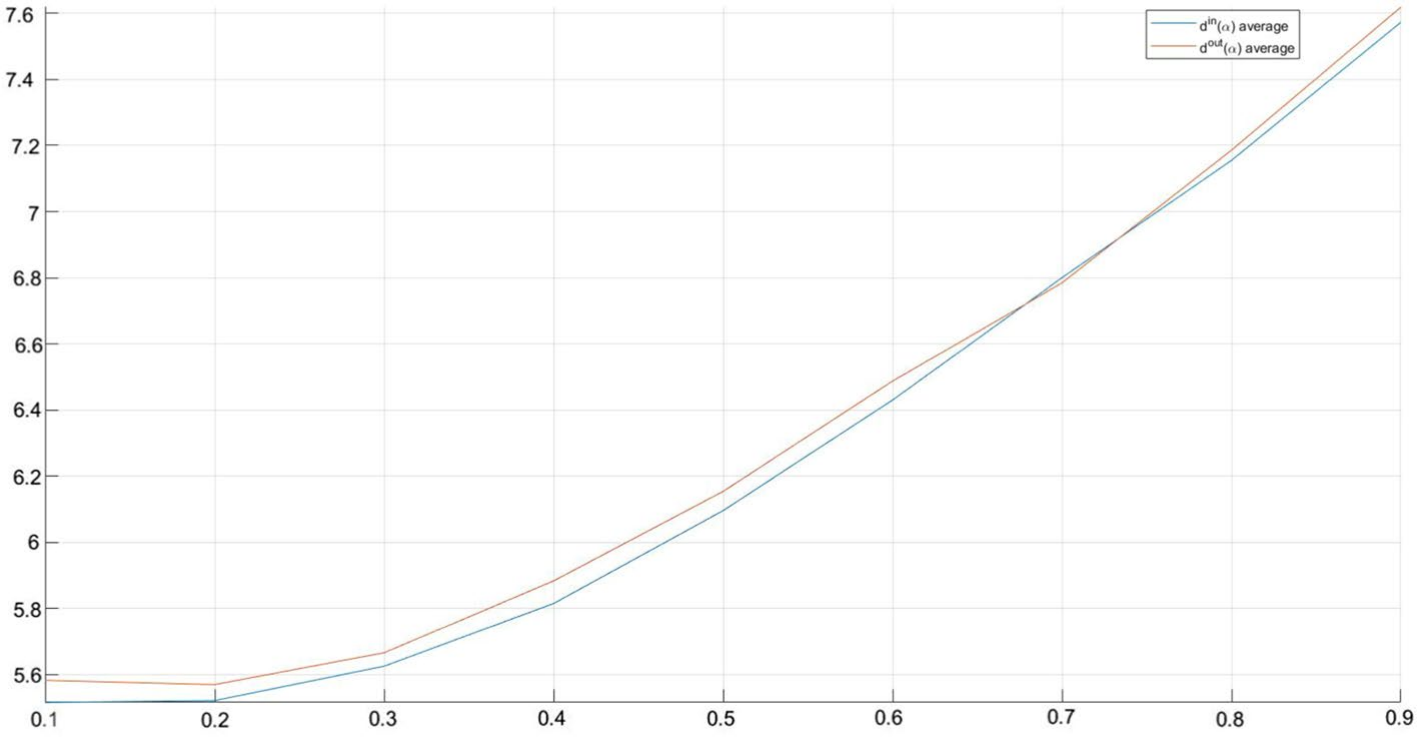
$$d^{in}(\alpha )$$ and $$d^{out}(\alpha )$$ means

In Fig. 5 there are the $d^{in}(\alpha )$ and $d^{out}(\alpha )$ means.

In Table 3 some descriptive statistics of $C^{in}(\alpha )$ and $C^{out}(\alpha )$ values are shown.

**Table 3:** Descriptive statistics of all the values contained in vectors $\mathbf{C }^{in}(\alpha )$ and $\mathbf{C }^{out}(\alpha )$

${{C}}^{in}$	0,1	0,2	0,3	0,4	0,5	0,6	0,7	0,8	0,9
Mean	0,0479	0,0476	0,0492	0,0515	0,0541	0,0575	0,0616	0,0660	0,0718
Median	0,0254	0,0255	0,0260	0,0269	0,0283	0,0307	0,0321	0,0352	0,0371
St.dev	0,0500	0,0493	0,0510	0,0542	0,0568	0,0602	0,0650	0,0680	0,0755
Max	0,0500	0,0493	0,0510	0,0542	0,0568	0,0602	0,0650	0,0680	0,0755
Min	0,0093	0,0097	0,0099	0,0108	0,0104	0,0114	0,0120	0,0113	0,0128
Skewness	1,5864	1,5902	1,5822	1,5852	1,5782	1,5904	1,5858	1,5703	1,5713
Kurtosis	3,6720	3,6984	3,6627	3,6649	3,6202	3,6845	3,6462	3,6026	3,5790

${{C}}^{out}$	0,1	0,2	0,3	0,4	0,5	0,6	0,7	0,8	0,9
Mean	0,0479	0,0476	0,0492	0,0515	0,0541	0,0575	0,0616	0,0660	0,0718
Median	0,0280	0,0285	0,0290	0,0304	0,0315	0,0339	0,0361	0,0393	0,0418
St.dev	0,0510	0,0501	0,0520	0,0555	0,0581	0,0611	0,0659	0,0695	0,0774
Max	0,2509	0,2491	0,2597	0,2949	0,3067	0,2973	0,3302	0,3525	0,3684
Min	0,0085	0,0086	0,0079	0,0094	0,0105	0,0113	0,0118	0,0115	0,0139
Skewness	2,2693	2,2502	2,2824	2,3297	2,3190	2,2556	2,3182	2,2855	2,3139
Kurtosis	7,6919	7,6007	7,8453	8,1035	8,0354	7,5954	7,9256	7,7954	7,8628

Summing up, it is possible to note that:

- in both cases of $C^{in} \alpha$ and $C^{out} \alpha$ the mean increases, slowly, when $\alpha$ increases. Similarly, the variability, always consistently high, increases with $\alpha$;
- maximum values in $C_{out}(\alpha )$ are greater then those in $C_{in}(\alpha )$, while the opposite result emerges for minimum values;
- the matrices $C^{in}(\alpha )$ show lower values for skewness and kurtosis with respect to matrices $C^{out}(\alpha )$. Both skewness and kurtosis values remain stable as $\alpha$ changes.

With reference to the borderline case we notice that the mean values are the same for $C^{in} (\alpha )$ and $C^{out} (\alpha )$ (and, of course, the same result holds for the standard deviation). Moreover, the dynamics of the mean are the same as the general case, with the only difference that the values change for a constant amount when $\alpha$ changes. The maximum and minimum values of $C^{in} (\alpha )$ are lower than those of $C^{out} (\alpha )$ and they increase as $\alpha$ changes. Skewness and kurtosis values are lower for $C^{in} (\alpha )$ and they remain quite stable both in $C^{in} (\alpha )$ and $C^{out} (\alpha )$. We notice that a customer that shows high $C^{in}$ value or high $C^{out}$ value maintains this characteristic for each value of $\alpha$.

**Fig. 6:**
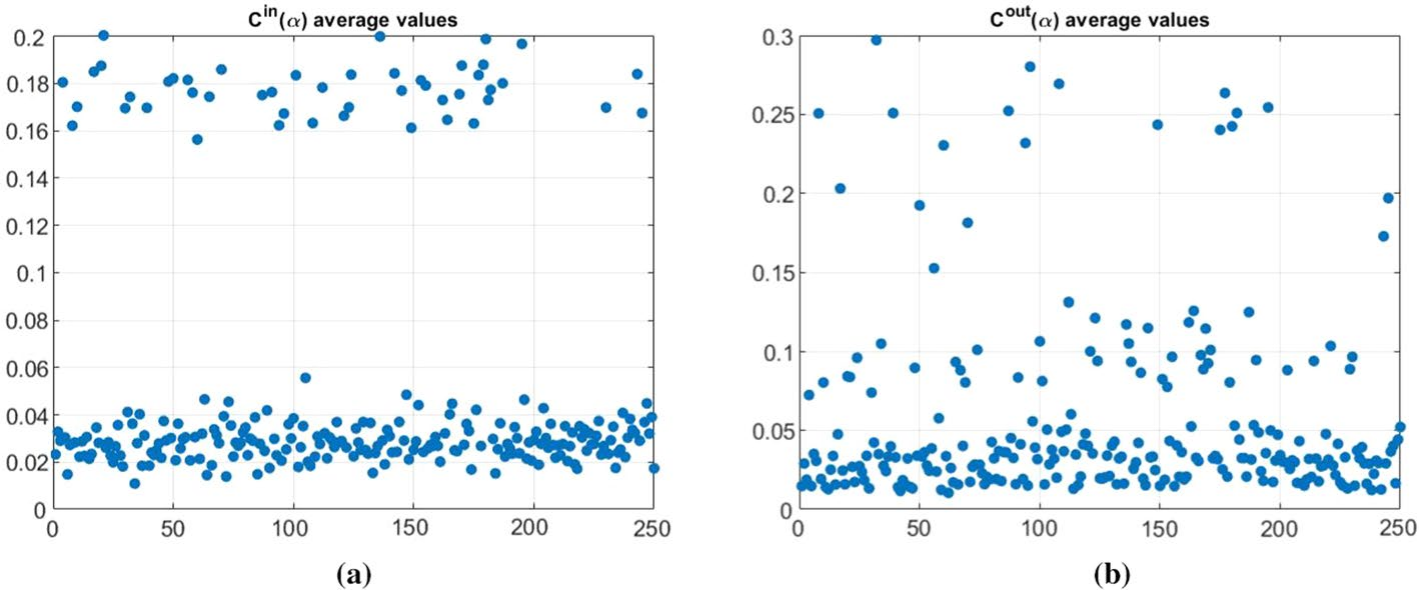
$C^{in}(\alpha )$ and $C^{out}(\alpha )$ mean values related to each customer firm

Stating the above, we synthesize the different $C^{in}$s and $C^{out}$s through the means of $C^{in}(\alpha )$ and $C^{out}(\alpha )$ over $\alpha =0,1, 0,2, \cdots , 0,9$, for each customer (see Fig. 6).

**Fig. 7:**
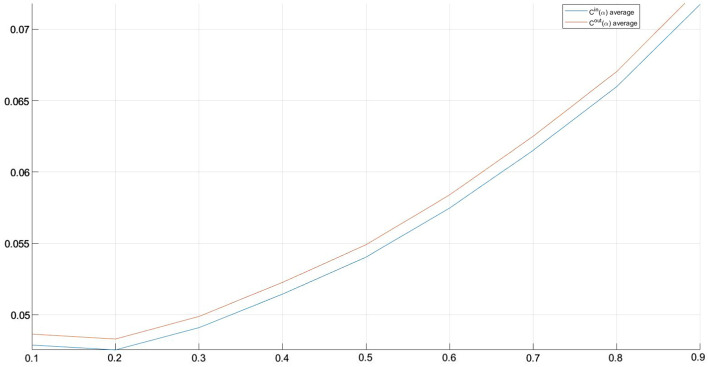
$$C^{in}(\alpha )$$ and $$C^{out}(\alpha )$$ means

In Fig. 7 there are the $C^{in}(\alpha )$ and $C^{out}(\alpha )$ means.

The $C^{in}$ and $C^{out}$ values obtained are quite low. It is thanks to these values that it is possible to assign an assessment to the quality of the credit granting policy adopted by the bank. In the specific case, the resilience analysis related to the specific structure of the network would seem to indicate an overall good practice of the bank in selecting the loan portfolio. In other words, it would seem that the system is capable of absorbing any specific difficulties of a company without causing particular damage to the other client companies belonging to the network.

Figure 6 allows highlighting, at a glance, those customers characterised by a stronger/weaker community structure around the nodes that represent them within the network. In particular, in the lower part of Figure 6 there are the customers that exhibit the weaker community structure. The customers that appeared effectively more vulnerable due to the behaviour of the other customers interconnected with them are 82.

For each customer belonging to a specific group, we now discuss the information regarding the economic sector, the legal form and the sales values, and some of the most crucial balance sheet ratios generally used during the creditworthiness investigation. In doing so, it is possible to search for the recurrence of specific characteristics among customers. Concerning the most vulnerable nodes, it is possible to note that there is no joint stock company among them and that the firms are equally distributed among the other legal forms (e.g., limited liability company $26\%$, limited partnership company $26\%$, general partnership $24\%$, sole proprietorship $24\%$)^9^.

It is interesting to note that, more frequently, the most vulnerable firms belong to the ’wholesale and retail trade’ ($27\%$), ’manufacture of textiles, of leather and related products and of wearing apparel’ ($39\%$), ’real estate and construction’ ($12\%$), ’arts and accomodation’ ($13\%$). Therefore, customers in such a group belong to economic sectors that have been particularly affected by the Covid-19 pandemic.

To analyse the relationships between the status of effectively vulnerable and the ratios provided by the bank, the values of these ratios have been grouped into three levels (low, medium and high)^10^. The firm size (measured through sales volume), EBITDA/sales and ROA would not seem linked to the status of effectively vulnerable. Indeed, these variables show a very low connection index that is also statistically significant (Cramer’s V equal to 0.07 - 0.20 - 0.41, respectively).

On the contrary, the link of the status of effectively vulnerable with leverage, ROE, ROI, the ratio credit drawn/credit granted, liquidity and default probability seems to be strong and significant (Cramer’s V equal to 0.64 - 0.57 - 0.53 - 0.59 - 0.74 - 0.50, respectively). In particular, more than half of vulnerable firms exhibit high leverage and high default probability as well as low levels of liquidity and profitability. These findings are therefore consistent with a framework of the substantial financial weakness of the customer firms considered.

Therefore, it seems that one has to add to the firms’ vulnerability caused by the negative impact of difficulties that affected other network nodes related to them another idiosyncratic source of vulnerability deriving from lousy management. This circumstance – which certainly exacerbates the weakness of the customer firms identified as vulnerable due to the spillover effect – should induce the bank to adequately reconsider these firms’ actual economic and financial health status. In particular, if needed, the bank should re-calibrate the terms related to the credit already granted (for example, by asking for the integration of guarantees) and adjust the risk coverage ratio through higher provisions.

The customers that appear really dangerous for the other nodes of the network due to the contagion effect are 98.

We notice that $53\%$ of these firms are limited liability companies. Similarly to what happened for effectively vulnerable customer firms, also the really dangerous firms belong more frequently to the ’manufacture of textiles, of leather and related products and of wearing apparel’ ($42\%$), ’wholesale and retail trade’ ($13\%$), ’accommodation and food service activities and arts, entertainment and recreation’ ($12\%$) and ’real estate activities and construction’ ($8\%$). Therefore, even in this case, we have the same sectors that were most affected by the impact of the Covid-19 pandemic on the Italian economy.

The link between the status of really dangerous and the other variables such as the firm’s size, the ratios EBITDA/sales, ROE, ROA and ROI turned out to be statistically significant but low (Cramer’s V equal to 0.16 – 0.15 – 0.18 – 0.13 – 0.15 respectively).

On the other hand, as already observed in the case of effectively vulnerable firms, the link between their status and other variables such as the leverage, the percentage of credit drawn/credit granted, the liquidity level and the default probability is statistically significant and moderate (Cramer’s V equal to 0.61 – 0.47 – 0.30 – 0.63 respectively). In particular, more than two-third of really dangerous firms exhibit a medium-high level of leverage, a medium-high percentage of credit drawn/credit granted, low levels of liquidity and very low levels of profitability. In contrast, more than half of these firms show a relatively high default probability. These figures are coherent with an overall situation of these kinds of bank customers firms’ financial weakness. Therefore, in addition to these firms’ dangerousness deriving from the network interconnection dynamics, it should also be considered the intrinsic danger derived from a not particularly solid financial profile. Also in this case, the bank should proceed with an adequate reconsideration of the actual economic and financial status of these firms and, if needed, re-calibrate the terms related to the credit already granted and an adjustment of the risk coverage ratio through higher provisions.

The customer firms in Group 1 contains 34 elements; Group 2 has 48 elements; Group 3 has 64 elements, while Group 4 has 104 elements.

We observe that the legal form is not a helpful element to distinguish the firms that belong to each group, as well as, the economic sector (indeed, the connection index is respectively about $41\%$ and $73\%$. On the contrary, it is pretty interesting to analyse the distribution of the Group 1 firms (i.e. the firms that the bank should carefully monitor because they appear to be both effectively vulnerable and really dangerous) with respect to the economic sector. Most of the Group 1 firms belong to sectors such as ’manufacture of textiles, of leather and related products and of wearing apparel’ ($44\%$), ’wholesale and retail trade’ ($18\%$), ’accommodation and food service activities and arts, entertainment and recreation’ ($18\%$) and ’real estate activities and construction’ ($21\%$).

The link between leverage and group type appears high (Cramer’s V equal to 0.73). In particular, all the firms in Group 1 exhibit high leverage, while the firms in group 4 exhibit low leverage. Similarly, the link between the group type and the percentage of credit drawn/credit granted is relatively high (Cramer’s V equal to 0.59). $62\%$ of Group 1 firms exhibit high percentages, while, on the opposite side, $94\%$ of the firms in Group 4 show a low percentage. Similarly, the liquidity index analysis reveals a strong link with the group typology (Cramer’s V equal to 0.56); all the firms in Group 1 show low liquidity levels, while all the firms in Group 4 exhibit medium-high liquidity levels. The link between default probability and group typology appears to be remarkable (Cramer’s V equal to 0.60). All the firms in group 4 exhibit low PD levels and $71\%$ of the firms in Group 1 show high levels.

These results are widely confirmed by the main descriptive statistics of the ratios above referred to the four groups of firms and presented in Table 4,^11^

**Table 4:** Descriptive statistics of the ratios for each group of firms

Typology	Mean	Median	Std. Dev.	Min	Max	cv
*Leverage*						
Group 1	2.86	2.92	0.23	2.43	3.19	0.08
Group 2	1.88	1.90	0.48	1.06	2.65	0.25
Group 3	1.80	1.91	1.07	0.07	3.48	0.59
Group 4	0.61	0.59	0.34	0.02	1.17	0.56
*EBITDA/sales*						
Group 1	0.06	0.05	0.04	0.00	0.12	0.68
Group 2	0.10	0.09	0.05	0.00	0.20	0.54
Group 3	0.11	0.11	0.05	0.00	0.22	0.45
Group 4	0.11	0.11	0.06	0.00	0.22	0.55
*ROE*						
Group 1	0.23	0.12	1.63	-2.16	6.44	7.16
Group 2	1.78	1.61	1.32	0.00	6.67	0.74
Group 3	5.54	4.12	4.16	0.21	13.48	0.75
Group 4	7.02	7.21	4.17	0.02	13.80	0.60
*ROA*						
Group 1	0.08	0.02	0.79	-1.16	3.52	10.53
Group 2	0.65	0.36	0.70	0.00	3.22	1.08
Group 3	2.93	1.44	3.20	0.00	12.12	1.09
Group 4	4.18	3.29	3.68	0.02	14.36	0.88
*ROI*						
Group 1	0.16	0.08	1.27	-1.54	5.48	7.84
Group 2	1.25	0.90	0.97	0.00	4.12	0.78
Group 3	4.13	2.99	3.29	0.15	11.00	0.80
Group 4	5.58	5.73	3.58	0.02	14.97	0.64
*Credit drawn/Credit granted*						
Group 1	0.91	0.93	0.15	0.64	1.15	0.17
Group 2	0.62	0.64	0.12	0.41	0.79	0.19
Group 3	0.54	0.48	0.19	0.30	0.96	0.34
Group 4	0.45	0.45	0.09	0.30	0.60	0.21
*Liquidity ratio*						
Group 1	0.25	0.24	0.08	0.10	0.49	0.33
Group 2	0.59	0.59	0.12	0.41	0.79	0.21
Group 3	0.92	0.89	0.31	0.41	1.90	0.34
Group 4	1.11	1.16	0.22	0.70	1.41	0.19
*Default Probability*						
Group 1	0.12	0.13	0.03	0.05	0.15	0.23
Group 2	0.10	0.13	0.05	0.01	0.15	0.48
Group 3	0.09	0.11	0.04	0.01	0.14	0.42
Group 4	0.03	0.03	0.01	0.01	0.05	0.45

## Conclusions

The work aims to suggest a methodology that a bank can employ to understand which customer firms to monitor carefully since, regardless of their health status, they show a high number and a strong intensity of reciprocal transactions and, therefore, a strong interdependence.

Therefore, the proposed approach allows a bank to benefit, in real-time, from capillary data about each customer ranging from traditional indices – coming from financial statements – to those of a more systemic nature underlying the dynamics that arise from the reciprocal interrelationships existing between its customers. Indeed, the bank can, at any time, combine the results relating to each customer and deriving from the proposed analysis methodology with others of a more traditional type based on the assessment of the values assumed by the most well-known accounting and management variables able to characterise the specific health status of each customer at that time.

In this respect, identifying the most vulnerable customers should suggest to the bank, as a precaution, to review the conditions of the credit granted to the former by carefully modulating the guarantees and, where possible, the pricing. On the contrary, the identification of a customer that is particularly dangerous for others should involve (i) the specific evaluation of the case and, therefore, the analysis of what could have been the causes, (ii) the consequent evaluation of the possibility to proceed with a review of the conditions applied to it and/or with higher provisions, as well as (iii) more careful monitoring of those customers that appear strongly related to it, even if they belong to the group of the customers neither vulnerable nor dangerous. Of course, the bank will prefer to manage with even greater attention all situations in which customers appear, at the same time, highly vulnerable to others and dangerous for others.

The methodology is remarkably versatile and can be applied to all the financial intermediation systems for which data are available; moreover, it can also be applied to banks characterized both by the same business model or by different ones. It is important to stress that our empirical instance is based on a local bank in northern Italy that provided all the required data. The high quality of the considered dataset is undoubtedly a further strength of this study.

Our approach could support bank managers, who could combine the proposed network-based methodology with the traditional credit risk models to improve default prediction, assess the bank’s loans portfolio’s quality, and the adequacy of its capital ratios and provisions.

From a macroscopic perspective, identifying the most critical bank’s customers offers a piece of intuitive information on the bank’s resilience as a system. Regulatory authorities could apply the same approach to study, for example, the dynamics emerging from the interbank flows network. As it is well known, this knowledge can help to understand the systemic risk entity and underlined forces during financial crises and distress and, as a consequence, to set the optimal level for capital requirements. In this respect, the presented methodology can be adopted by a central bank with respect to the interbank market in order to identify critical nodes (banks) thanks to the knowledge of the interconnections between the participants to the same market.

The empirical network analysed in this work comprises only the firms that are customers of the same bank and have reciprocal relationships. For this reason, the network does not take into consideration the incoming or outgoing flows with other subjects that do not belong to the same system, as happens in the real world. Thus, we do not consider the contributions to the network of the connections with firms outside it. In this respect, the flows exchanged with the other external counterparties that we did not consider could act as a bearing absorbing specific difficulties. Therefore, the analysis of a more complex environment – where the action of the entities outside the network can be opportunely measured – can be a challenging theme for future research.

In this respect, it would be interesting to develop a model based on neural networks (for a survey on neural networks, see e.g. Gurney 2018) for discussing the dynamics of systemic risk. In particular, one can elaborate on how the interaction of the firms evolves over time and reorganizes itself by employing the information on the past. In so doing, one should introduce a learning phase to transform an initial input on shock propagation to a final output. Such a challenging theme deserves a devoted research project.

The network topology knowledge is also helpful to discover whether there are regularities between particular types of customers to be included at the initial credit granting assessment. It is also helpful to further improve the level of banking services and customer satisfaction and to establish the foundation for more in-depth study of customer trading network structure from the micro-level too. Eventually, if the bank knows the network structure, it can carry out scenarios analysis to anticipate shocks and, as a consequence, set the best strategy to manage the customers’ relationships.

Finally, research on topological structural characteristics may guide the bank to optimise business patterns, transform complex customer management to plane organised network management, and acquire new customer resources to raise the bank’s reputation.

## Footnotes

1 See Hand and Henley (1997) and Thomas (2009) for a complete review of the main statistical modelling approaches to credit scoring.

2 We thank the bank that provided the data and let us develop the analysis. The name of the bank cannot be given for reasons of confidentiality.

3 For a complete review of the models that exploit the benefits of relationship information in banking engagement, see (Boot 2000) and (Agarwal et al. 2009).

4 See, e.g., Khandani et al. (2010), where the authors apply machine learning to obtain nonlinear parametric forecasting models of consumer credit risk starting from banking transactions data and credit bureau data. Butaru et al. (2016) use account-level credit card data from a bank, credit bureau data and macroeconomic data to predict delinquency. Galindo and Tamayo (2000) use machine learning (CART decision tree models) on mortgage loan data to detect defaults. Huang et al. (2004) offer a survey of corporate credit rating models and show that artificial intelligence models have a better performance than traditional statistical models.

5 See Petropoulos et al. (2019)

6 The resilience of a network (or its fragility) represents its capability of survival after the failure of some of its nodes and it is recognized as an important chapter in the theory of complex networks (Albert and Barabási 2002).

7 Data available upon request.

8 Data available upon request.

9 As an example, if a particular legal form had revealed to be more frequent, the bank could have verified, for instance, whether this was due to disadvantageous situations deriving from regulations impacting this specific category of firms.

10 Since the balance sheet ratio distributions are skewed, as usual, we split the values of the ratio into three equally sized intervals.

11 We ran the well-known statistical tests on the consider dataset to verify the real difference between the average values. The results let us reject the null hypothesis of no difference between the group averages with a significance level of 99$\%$.

## References

[CR1] Addo PM, Guegan D, Hassani B (2018). Credit risk analysis using machine and deep learning models. Risks.

[CR2] Agarwal S, Skiba PM, Tobacman J (2009). Payday loans and credit cards: new liquidity and credit scoring puzzles?. Am Econom Rev.

[CR3] Albert R, Barabási A-L (2002). Statistical mechanics of complex networks. Rev Modern Phys.

[CR4] Allen F, Carletti E (2013). What is systemic risk?. J Money, Credit and Bank.

[CR5] Allen F, Gale D (2000). Financial contagion. J Political Econom.

[CR6] Altman EI (1980) Commercial bank lending: process, credit scoring, and costs of errors in lending. J Financial and Quantitative Anal, pp. 813–832

[CR7] Aymanns C, Georg C-P (2015). Contagious synchronization and endogenous network formation in financial networks. J Bank & Finance.

[CR8] Biswas SS, Gómez F (2018). Contagion through common borrowers. J Financial Stabil.

[CR9] Boot A (2000) Relationship banking: What do we know? J Financial Intermed-tion, 9

[CR10] Butaru F, Chen Q, Clark B, Das S, Lo AW, Siddique A (2016). Risk and risk management in the credit card industry. J Bank & Finance.

[CR11] Castiglionesi F (2007). Financial contagion and the role of the central bank. J Bank & Finance.

[CR12] Cernov M, Urbano T (2018) Identification of eu bank business models. EBA Staff Paper Series

[CR13] Cerqueti R, Clemente GP, Grassi R (2021). Stratified cohesiveness in complex business networks. J Bus Res.

[CR14] Chen MC, Huang SH (2003). Credit scoring and rejected instances reassigning through evolutionary computation techniques. Exp Syst Appl.

[CR15] Chen TK, Liao HH (2018). Suppliers’/customers’ production efficiency uncertainty and firm credit risk. Rev Quan Finance and Account.

[CR16] Chinazzi M, Fagiolo G (2015) Systemic risk, contagion, and financial networks: a survey. Institute of Economics, Scuola Superiore Sant’Anna, Laboratory of Economics and Management (LEM) Working Paper Series(2013/08)

[CR17] Clemente GP, Grassi R (2018). Directed clustering in weighted networks: a new perspective. Chaos, Solitons & Fractals.

[CR18] Duffie D, Eckner A, Horel G, Saita L (2009). Frailty correlated default. J Finance.

[CR19] Edirisinghe C, Gupta A, Roth W (2015). Risk assessment based on the analysis of the impact of contagion ow. J Bank & Finance.

[CR20] Eshleman JD, Guo P (2014). The market’s use of supplier earnings information to value customers. Rev Quan Finance and Account.

[CR21] Gai P, Haldane A, Kapadia S (2011). Complexity, concentration and contagion. J Monetary Econom.

[CR22] Galindo J, Tamayo P (2000). Credit risk assessment using statistical and machine learning: basic methodology and risk modeling applications. Comput Econom.

[CR23] Gençcay R, Pang H, Tseng MC, Xue Y (2020) Contagion in a network of heterogeneous banks. J Bank & Finance 111:105725

[CR24] Giesecke K (2004) Credit risk modeling and valuation: an introduction. Available at SSRN 479323

[CR25] Giesecke K, Weber S (2004). Cyclical correlations, credit contagion, and portfolio losses. J Bank & Finance.

[CR26] Giesecke K, Weber S (2006). Credit contagion and aggregate losses. J Econom Dynam Control.

[CR27] Giudici P, Hadji-Misheva B, Spelta A (2019). Network based scoring models to improve credit risk management in peer to peer lending platforms. Front Artificial Intell.

[CR28] Glasserman P, Young HP (2015). How likely is contagion in financial networks?. J Bank & Finance.

[CR29] Goldstein RS, Helwege J, Cantor, WTR, Das S, Lee K, Fedak A (2002). Are jumps in corporate bond yields priced? modeling contagion via the updating of beliefs. working paper

[CR30] Gurney K (2018). An introduction to neural networks.

[CR31] Hand DJ (1981) Discrimination and classification. Wiley Series in Probability and Mathe-matical Statistics

[CR32] Hand DJ, Henley WE (1997). Statistical classification methods in consumer credit scoring: a review. J Royal Statistical Soc: Series A (Statistics in Society).

[CR33] Hatchett JP, Kuehn R (2009). Credit contagion and credit risk. Quan Finance.

[CR34] Heinsalu E, Pampurini F, Patriarca M, Quaranta AG (2020) Network resilience and assessment of the credit granting policy. International Review of Business Research Papers, 16 (2)

[CR35] Horst U (2007). Stochastic cascades, credit contagion, and large portfolio losses. J Econom Behav Organiz.

[CR36] Huang Z, Chen H, Hsu C-J, Chen W-H, Wu S (2004). Credit rating analysis with support vector machines and neural networks: a market comparative study. Decision Support Syst.

[CR37] Jarrow RA, Yu F (2001). Counterparty risk and the pricing of defaultable securities. J Finance.

[CR38] Johnson R, Wichern D (1998). Principal components. Applied Multivariate Statistical. Analysis.

[CR39] Jorion P, Zhang G (2007). Good and bad credit contagion: evidence from credit default swaps. J Financial Econom.

[CR40] Kahya E, Panayiotis T (1999). Predicting corporate financial distress: a time-series cusum methodology. Rev Quan Finance and Account.

[CR41] Khandani AE, Kim AJ, Lo AW (2010). Consumer credit-risk models via machinelearning algorithms. J Bank Finance.

[CR42] Lachenbruch PA, Goldstein M (1979). Discriminant analysis. Biometrics.

[CR43] Lacher RC, Coats PK, Sharma SC, Fant LF (1995). A neural network for classifying the financial health of a firm. Eur J Operat Res.

[CR44] Martinez-Jaramillo S, Battiston S (2020). Network models and stress testing for financial stability: The conference. J Financial Stabil.

[CR45] Merton RC (1974). On the pricing of corporate debt: the risk structure of interest rates. J Finance.

[CR46] Newman ME (2003). The structure and function of complex networks. SIAM Rev.

[CR47] O’Brien M, Dyché J et al. (2002) The crm handbook: A business guide to customer relationship management. Addison-Wesley Professional

[CR48] Onnela J-P, Saramäki J, Kertész J, Kaski K (2005). Intensity and coherence of motifs in weighted complex networks. Phys Rev E.

[CR49] Petrone D, Latora V (2018). A dynamic approach merging network theory and credit risk techniques to assess systemic risk in financial networks. Sci Rep.

[CR50] Petropoulos A, Siakoulis V, Stavroulakis E, Klamargias A et al. (2019) A robust machine learning approach for credit risk analysis of large loan level datasets using deep learning and extreme gradient boosting. IFC Bulletins chapters, 49

[CR51] Pino G, Sharma S (2019). On the contagion effect in the us banking sector. J Money, Credit and Bank.

[CR52] Pu X, Zhao X (2012). Correlation in credit risk changes. J Bank & Finance.

[CR53] Roengpitya R, Tarashev N, Tsatsaronis K, Villegas A (2014) Bank business models. BIS Quarterly Review, December

[CR54] Roengpitya R, Tarashev N, Tsatsaronis K, Villegas A (2017) Bank business models: Popularity and performance. BIS Working Papers, p. 682

[CR55] Saramäki J, Kivelä M, Onnela J-P, Kaski K, Kertesz J (2007). Generalizations of the clustering coefficient to weighted complex networks. Phys Rev E.

[CR56] Schönbucher PJ, Schubert D (2001) Copula-dependent default risk in intensity models. In Working paper, department of statistics, bonn university

[CR57] Setiono R, Thong JY, Yap C-S (1998). Symbolic rule extraction from neural networks: an application to identifying organizations adopting it. Inform Manag.

[CR58] Shin HS (2010) Financial intermediation and the post-crisis financial system

[CR59] Thomas DA (2009). Mapping your network. Harvard Business School Organizational Behavior Unit

[CR60] Trustorff J-H, Konrad PM, Leker J (2011). Credit risk prediction using support vector machines. Rev Quan Finance and Account.

[CR61] West D (2000). Neural network credit scoring models. Comput Operat Res.

